# Esculetin induces antiproliferative and apoptotic response in pancreatic cancer cells by directly binding to KEAP1

**DOI:** 10.1186/s12943-016-0550-2

**Published:** 2016-10-18

**Authors:** Rashi Arora, Sharad Sawney, Vikas Saini, Chris Steffi, Manisha Tiwari, Daman Saluja

**Affiliations:** Dr. B.R. Ambedkar Centre for Biomedical Research, University of Delhi, Delhi, 110007 India

**Keywords:** Esculetin, Coumarins, Molecular target, Pancreatic cancer, Nrf2, KEAP1, NF-κB, ARE pathway, Anticancer compound

## Abstract

**Background:**

A handful of studies have exploited antitumor potential of esculetin, a dihydroxy coumarine derivative; the targets to which it binds and the possible downstream mechanism for its cytotoxicity in cancer cells remain to be elucidated. Using pancreatic cancer cell lines as a model system, herein the study was initiated to check the efficacy of esculetin in inhibiting growth of these cancer cells, to decipher mechanism of its action and to predict its direct binding target protein.

**Methods:**

The cytotoxicity of esculetin was determined in PANC-1, MIA PaCa-2 and AsPC-1 cell lines; followed by an inspection of intracellular levels of ROS and its associated transcription factor, p65-NF-κB. The interaction between transcription factor, Nrf2 and its regulator KEAP1 was studied in the presence and absence of esculetin. The effect of Nrf2 on gene expression of antioxidant response element pathway was monitored by real time PCR. Thereafter, potential binding target of esculetin was predicted through molecular docking and then confirmed in vitro.

**Results:**

Esculetin treatment in all three pancreatic cancer cell lines resulted in significant growth inhibition with G1-phase cell cycle arrest and induction of mitochondrial dependent apoptosis through activation of caspases 3, 8 and 9. A notable decrease was observed in intracellular ROS and protein levels of p65-NF-κB in PANC-1 cells on esculetin treatment. Antioxidant response regulator Nrf2 has been reportedly involved in crosstalk with NF-κB. Interaction between Nrf2 and KEAP1 was found to be lost upon esculetin treatment in PANC-1 and MIA Paca-2 cells. Nuclear accumulation of Nrf2 and an upregulation of expression of Nrf2 regulated gene NQO1, observed on esculetin treatment in PANC-1 further supported the activation of Nrf2. To account for the loss of Nrf2-KEAP1 interaction on esculetin treatment, direct binding potential between esculetin and KEAP1 was depicted *in silico* using molecular docking studies. Pull down assay using esculetin conjugated sepharose beads confirmed the binding between esculetin and KEAP1.

**Conclusions:**

We propose that esculetin binds to KEAP1 and inhibits its interaction with Nrf2 in pancreatic cancer cells. This thereby promotes nuclear accumulation of Nrf2 in PANC-1 cells that induces antiproliferative and apoptotic response possibly by attenuating NF-κB.

**Electronic supplementary material:**

The online version of this article (doi:10.1186/s12943-016-0550-2) contains supplementary material, which is available to authorized users.

## Background

Naturally occurring compounds have provided numerous health benefits to human race since time immemorial. A plethora of compounds have been under rigorous screening for anticancer potential and developed as chemotherapeutic agents [1, 2]. Amongst the coumarins, 6,7-dihydroxy derivative, commonly called esculetin, was reported to induce antiproliferative response in several cancer cell lines [3]. It is found in various medicinal plants such as *Cichorium intybus* (Asteracea)*, Artemisia capillaries* (Compositae), *Ceratostigma willmottianum* (Plumbaginaceae), *Citrus limonia* (Rutaceae) etc [4, 5]. Several reports have presented induction of apoptosis and cell cycle arrest in human cancer cells on treatment with esculetin [6–14]. Although these studies present an insight into various signaling pathways that get misregulated on esculetin treatment, the direct target(s) of the compound remains to be elucidated. Further, esculetin is a well established antioxidant [15], and thus antioxidant responsive pathway merits attention.

Nuclear Factor-Erythroid 2-related factor 2 (Nrf2), encoded by Nuclear Factor-Erythroid 2-like2 (*NFE2L2*) gene is a major regulator of antioxidant response in cell [16]. It is a basic region-leucine zipper (bZIP) protein belonging to Cap ‘n’ Collar (CNC) family of transcription factors and binds to Antioxidant Response Element (ARE) present in promoter of a battery of genes coding for antioxidant proteins [17]. Nrf2 signaling remains under tight regulation by an actin binding protein, Kelch-like ECH-associated protein1 (KEAP1) [18]. Under normal conditions KEAP1 sequesters Nrf2 in cytoplasm and promotes its ubiquitination and proteosomal degradation [19]. Owing to a number of cysteine residues present throughout the KEAP1 protein, specifically Cys151, Cys273 and Cys288, it senses oxidative stress and thereby liberates Nrf2, which then gets accumulated in nucleus and activates transcription of genes under the ARE regulation [20].

Role of Reactive Oxygen Species (ROS) in cancer progression and treatment has always remained debatable. Over the past several years, it has been established that cancer cells have far greater ROS levels than normal cells and that ROS mediated regulation of transcription factors like nuclear factor kappa-light-chain-enhancer of activated B cells (NF-κB) etc are required for tumorigenesis, cancer cell survival, proliferation and metastasis [21]. Considerable attention has therefore been given to developing ROS depleting strategies to vanquish tumor growth. Recently, activation of Nrf2 mediated ARE signaling has emerged as an attractive strategy for treatment of cancer [22, 23]. Consequently, small molecules that inhibit Nrf2-KEAP1 interaction are springing up as promising chemotherapeutic agents for cancer.

Using pancreatic cancer cells (PANC-1; MIA PaCa-2 and AsPC-1) as a model system, herein we first tried to look into the potential of esculetin as an antiproliferative and apoptotic agent. To elucidate the mechanism of action of esculetin, cellular ROS status and level of NF-κB was examined in PANC-1 cells on esculetin treatment. Thereafter we investigated if the effect is mediated through disruption of Nrf2-KEAP1 interaction. Finally, we deciphered the molecular target to which esculetin possibly binds by examining its binding potential with KEAP1 through computer based molecular docking and subsequently validated this interaction in vitro. We have also examined pharmacokinetics of esculetin by using *in silico* tools.

## Methods

### Compound

Esculetin (6,7-dihydroxycoumarin, 98 % purity) was purchased from Sigma-Aldrich (USA) and dissolved in dimethyl sulfoxide (DMSO, vehicle).

### Cell culture

Human embryonic kidney cells – HEK 293 and Human pancreatic carcinoma cells- PANC-1, MIA PaCa-2 and AsPC-1 were cultured in high glucose Dulbecco minimal essential medium (DMEM) (Sigma-Aldrich, USA) supplemented with 10 % fetal bovine serum (GIBCO, USA) and penicillin (60 IU/ml) / streptomycin (50 μg/ml) at 37 °C in 5 % CO_2_ humidified atmosphere. The HEK 293 cell line was provided as a kind gift by Prof Vani Brahmachari and all the three pancreatic cell lines were a kind gift from Dr Madhu Chopra, Dr. B.R Ambedkar Centre for Biomedical Research, University of Delhi.

### Cell viability

The cells were grown up to 70 % confluency in 96 well plates and treated with different concentration of esculetin for the indicated time. Control cells were supplemented with complete media containing 0.1 % DMSO (vehicle control) for various time points. MTT solution [0.5 % (v/v)] was prepared in Phosphate Buffer Saline (PBS) and 20 μl of the solution was added to each well. After incubation at 37 °C for 4 h, DMSO was added to each well to dissolve formazan crystals. Absorbance of each well was measured at 570 nm in ELISA plate reader (Tecan, Grӧdlg, Austria) and percentage of cell viability was calculated with respect to vehicle control (VC).

### Cell cycle analysis

The cells at 60 % confluency in T-25 flasks were deprived of serum for 24 h. The G0 phase synchronous population of cells thus obtained was treated with 100 μM of esculetin for different time points. After the desired time interval, cells were washed with cold PBS, centrifuged and fixed in 70 % (v/v) ethanol at 4 °C. Ethanol was then removed by washing the cells twice with cold PBS. Cells were then incubated in PBS containing RNaseA (0.005 mg/ml) at 37 °C for 30 min. Subsequently, Propidium Iodide (PI) (0.1 mg/ml) was added to the cells and incubated at room temperature for 15 min in dark. The cells were then analysed for their distribution in different phases of cell cycle on FACScalibur using CellQuestPro software (Becton Dickinson, USA).

### Detection of apoptosis by Annexin-V and Propidium Iodide (PI) staining

Annexin V, an apoptotic marker binds to phosphatidylserine (PS) that translocates from the inner membrane of the plasma membrane to the outer membrane during apoptosis and thus gives a measure of the percentage of cells actively undergoing apoptosis. PI on the other hand gives a measure of cell viability as cells with intact cell membrane exclude PI. Cells were treated with 100 μM esculetin for different time points. They were then stained with allophycocyanin (APC) labeled annexin-V and PI as per the manufacturer’s guidelines (eBiosciences,USA). Population was then analyzed for percentage of cells in healthy, early apoptotic and late apoptotic phase on FACScalibur using CellQuestPro software.

### Detection of apoptosis by terminal deoxynucleotidyltransferase dUTP nick end labeling (TUNEL) assay

DNA fragmentation by endonucleases is one of the processes that set in during apoptosis resulting in creation of multiple 3′hydroxyl ends. These ends are labeled in APO-BRDU™ assay with bromo-deoxyuridine triphosphates (Br-dUTP) using terminal deoxynucleotedyl transferase (TdT) and are identified by flow cytometry by staining the cells with a FITC-labeled anti-BrdU mAb. Staining was done as per the manufacturer’s guidelines (BD Pharmingen) and analyzed on FACScalibur using CellQuestPro software.

### Mitochondrial membrane potential assay

Mitochondrial membrane potential was determined in untreated cells and cells treated with 100 μM esculetin for different time using MitoProbe JC-1 assay kit (Life technologies, USA). JC-1, a mitochondrial membrane potential sensor, is a lipophilic cationic dye that exists as aggregates in healthy cells and emits red fluorescence. Whereas, once mitochondrial membrane gets depolarized it exists as monomers in cytoplasm and emit green fluorescence. Ratio of red and green fluorescence signals from cells stained with JC-1 dye was recorded and analyzed on FACScalibur using CellQuestPro software.

### Western blot analysis

Cells cultured in T-25 flasks were treated with 100 μM esculetin for different time intervals. They were lysed in RIPA buffer (50 mM Tris, 2 mM EDTA, 150 mM NaCl, 1 % Tergitol, 0.5 % sodium deoxycholate, 1 mM PMSF and 1X Protease Inhibitor) for 45 min at 4 °C and centrifugated at 13,000 g for 20 min at 4 °C. Total protein obtained as supernatant was determined using BCA protein estimation kit (Bangalore Genei). For collection of separate nuclear and cytoplasmic extract, NE-PER™ Nuclear and Cytoplasmic Extraction kit (Life Technologies, USA) was used as per manufacturer’s protocol. Equal amount of proteins (30–50 μg) were boiled in Laemmli buffer (2 % SDS, 10 % glycerol, 60 mM Tris-Cl pH 6.8, bromophenol blue 0.02 %) and separated on (8–12 %) SDS- polyacrylamide gels (SDS-PAGE) and transferred on polyvinyldifluoride (PVDF) membrane (Millipore, USA). Bovine Serum Albumin (BSA), 5 %, was used to block the PVDF membrane, followed by incubation with desired primary antibody for 3 h and horseradish peroxidase (HRP) conjugated secondary antibody for 45 min at 37 °C with intermittent washing thrice with 0.05 % tween-20 in PBS (v/v) at room temperature for 15 min each. Immunoreactive bands were probed with the enhanced chemiluminiscence (ECL) western blot detection system (Biogene, India) according to manufacturer’s instructions and viewed in gel documentation system LAS4000 (FUJIFILM, USA). Antibodies against caspase 3 (MA191637), caspase 8 (MA141280), caspase 9 (MA112562) and cytochrome C (MA5-11674) were purchased from Pierce (Thermo Scientific, USA). Antibody against phosphorylated Nrf2 (ab76026) was purchased from abcam. Antibody against proliferating cell nuclear antigen (PCNA) (ab18197) and NF-κB (sc-372) were provided as a kind gift by Prof Vani Brahmachari and Prof K Natrajan respectively from Dr. B.R Ambedkar Centre for Biomedical Research, University of Delhi. Antibody against inhibitor kappa B (IκB) (sc-847) was a kind gift by Prof Alok C Bharti from Department of Zoology, University of Delhi. Antibody against β actin (sc-47778), Nrf2 (sc-13032) and KEAP1 (sc-15246) along with all HRP conjugated secondary antibodies against rabbit, mouse and goat were purchased from Santa Cruz (USA).

### Measurement of intracellular ROS level

Cells treated with 100 μM esculetin were incubated with 10 μM of 2′,7′-Dichlorofluorescin diacetate (DCFDA) at 37 ^o^C for half an hour. This cell-permeable non-fluorescent probe gets oxidized to highly fluorescent 2′,7′-dichlorofluorescein upon exposure to ROS. The cells were then washed with PBS and analyzed on FACScalibur using CellQuestPro software.

### Co-immunoprecipitation

Untreated and esculetin (100 μM) treated cells were collected and lysed in buffer (20 mM Tris, 0.5 mM EDTA, 100 mM NaCl, 0.5 % Tergitol, 1 mM PMSF and 1X Protease Inhibitor). Equal amount of soluble protein (0.5 mg) from treated and untreated samples were allowed to immunoprecipitate overnight with 2 μg of desired antibody at 4 °C. The immunocomplex was then allowed to bind to 40 μl of Protein A/G agarose beads (SantaCruz, USA) and complex was collected by centrifugation at 1500 rpm for 5 mins at 4 °C and washed thrice in lysis buffer. The complex was then eluted by boiling in SDS-Laemmli buffer with β mercaptoethanol and fractionated on 10 % SDS-PAGE, followed by western blot analysis for the desired protein. Equal amount of input (10 % of total protein used in Co-IP) was used as endogenous control.

### Confocal microscopy

Cells were grown on coverslips placed in 6-well plate and treated with 100 μM esculetin. Treated cells were fixed with 2 % PFA (Paraformaldehyde) at room temperature for 15 mins. Fixed cells were permeablized with perm buffer (0.2 % saponin and 0.1 % BSA), blocked with 5 % BSA and incubated overnight in anti-Nrf2 antibody at 4 °C and then in fluorescein (FITC) conjugated secondary antibody for 2 h at room temperature. After washing the cells, they were fixed with 4 % PFA and stained with nuclear stain DAPI (4′,6-diamidino-2-phenylindole). Confocal imaging was performed with Nikon C2 laser scan confocal microscope with 60× objective magnification, numerical aperture 1.4, refractive index 1.5, Plan Apo optics equipped with an argon laser, using excitation and emission wavelength of 490 and 525 nm respectively for FITC and 461 and 500 nm respectively for nuclear stain DAPI (4′,6-diamidino-2-phenylindole). Data was analyzed using the NIS Elements AR software.

### RNA purification, cDNA synthesis and qPCR

RNA was isolated from esculetin treated and untreated cell cultures using RNeasy kit (Qiagen, Netherlands) according to manufacturer’s instructions. cDNA was synthesized from 1 μg of RNA using First strand cDNA synthesis kit (Thermo Scientific, USA) according to the manufacturer’s protocol. Expression of NAD(P)H:quinone oxidoreductase 1 (*NQO1*) gene was measured by carrying out real-time PCR reactions using gene specific primers on cDNA so prepared. Reaction mix constituted 1X SYBR green mix (ROCHE, Switzerland), template cDNA and 0.5 μM primers and PCR was carried out on Applied Biosystems 7300 RT-PCR system (Life technologies, USA). Data was collected using SDS 2.2.2 version and relative expression was calculated using 2^-ΔΔCT^ method with GAPDH (Glyceraldehyde 3-phosphate dehydrogenase) as housekeeping control. Following primers were used: 

NQO1qFP: 5′ GGCAGAAGAGCACTGATCGTA 3′

NQO1qRP: 5′-TGATGGGATTGAAGTTCATGGC-3′

GAPDH FP: 5′ CAAGGTCATCCATGACAACTTTG 3′

GAPDH RP: 5′ GTCCACCACCCTGTTGCTGTAG 3′

### Docking studies

Docking experiments were performed using Discovery Studio (DS) Client v4.0 package (Accelrys Inc., USA). The ligand molecule, esculetin, was constructed using the Build Fragment tool in DS. Hydrogen atoms were added and valency was monitored followed by the use of clean geometry application. The ligand was applied with CHARMm force field, followed by energy minimization using steepest descent and conjugate gradient methods till a derivative of 0.001 is achieved. The coordinates for the X-ray crystal structures of the receptor were obtained from the RCSB protein data bank (PDB ID: 4IQK). For docking studies, initial protein was prepared by removing all water molecules, heteroatoms, any co-crystallized solvent and the ligand. Proper bonds, bond orders, hybridization and charges were assigned using protein modelling tools followed by application of CHARMm force field. The ligand was removed and a site sphere was specified to define the active site of KEAP1 receptor. Other than KEAP1, modified KEAP1 receptors were also used wherein the amino acids Arg 415 and Arg 483 were replaced by Alanine one by one. The CDOCKER program of DS 4.0 software was used to perform docking simulations and score ligand pose module was performed to determine binding affinity scores. CDOCKER generates random conformations of ligands within the active site through high-temperature molecular dynamics to generate 10 docked ligand poses. The docking accuracy was evaluated in terms of the root mean square deviation (RMSD), lying within 2.0 Å between the docked position and the experimentally determined position for the ligand. Further, the stability of enzyme-ligand complex was observed, based on various scoring function such as Lig score, PLP, PMF, LUDI and CDOCKER interaction energy.

### Cell based pull-down assay

CNBr-activated Sepharose 4B beads (GE Healthcare) were coupled with esculetin as described by Lee *et al* [24]. Coupled and uncoupled beads were separately incubated overnight with total cellular protein extract in reaction buffer [50 mM Tris (pH7.5), 5 mM EDTA, 150 mM NaCl, 1 mM DTT, 0.01 % Tergitol, 0.2 % BSA, 0.2 mM PMSF and 1X protease inhibitor cocktail]. Beads were washed thrice with reaction buffer followed by elution of retained protein in laemmli buffer with β mercaptoethanol. The protein was then fractionated on 10 % SDS-PAGE and checked for presence of KEAP1 by western blotting. Further, to assert the specificity of binding, competition assay was carried out by pre-incubating total cellular protein with variable concentrations of esculetin and subsequently using this protein mixture for pull down assay as described above.

### ADME property prediction

ADME (Absorption, Distribution, Metabolism and excretion) properties of esculetin were predicted using ADMET module, v. 4.0 (Accelrys Inc., USA). This gave an estimate of the physicochemical properties and the bioavailability of the compounds. Parameters such as polar surface area (PSA), CYP2D6 (cytochrome P450 2D6 binding), hepatotoxicity, absorption level (Human intestinal absorption), and PPB (plasma protein binding), BBB (Predicted brain/blood partition coefficient), and solubility level (Predicted aqueous solubility) were calculated. The acceptability of esculetin based on the Lipinski’s rule of five [25] was also estimated from the results.

### Statistical analysis

All data is represented as mean ± SD of three independent experiments. Differences between the means were determined using one-way analysis of variance (ANOVA). A value of *p* < 0.05 was set as the level of significance.

## Results

### Esculetin induces cytotoxicity and inhibits proliferation of pancreatic cancer cells

**Fig. 1:**
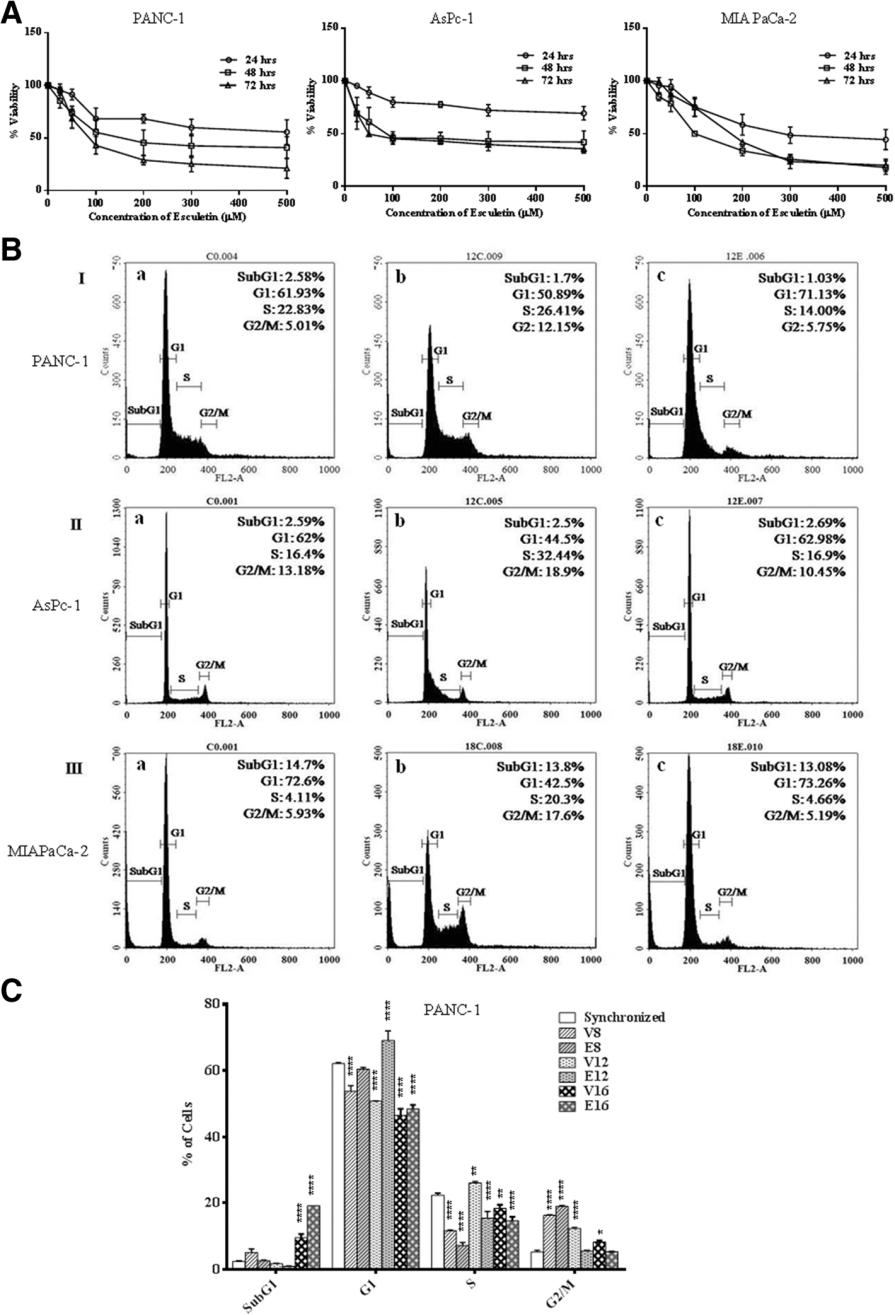
Effect of esculetin on pancreatic cancer cells: **A** Effect of different concentrations of esculetin on cell viability using MTT assay in PANC-1, AsPc-1 and MIA PaCa-2 cell lines. **B** Cell cycle analysis of different cell lines ((I) PANC-1 (II) AsPc-1 and (III) MIA PaCa-2) in the absence and presence of esculetin (100 μM) using flow cytometric analysis of DNA content showing cell cycle arrest in G1 phase (a-synchronized population, b-vehicle control, c- esculetin treated cells for 12 h in I and II and 18 h in III). **C** Percent distribution of PANC-1 cells in different phases of cell cycle upon 100 μM esculetin treatment. (V stands for Vehicle control for indicated time, E stands for esculetin treated sample for indicated time). Data represents the mean ± SD of three independent experiments. The significance was determined using ANOVA (Bonferroni’s test). Key:*p < 0.05; **p < 0.01; ***p < 0.001; **** *p* < 0.0001)

Effect of esculetin on cell viability was determined on three different pancreatic cancer cell lines- PANC-1, MIA PaCa-2 and AsPC-1 and on HEK 293 as a control by MTT assay. It was found to inhibit the growth of all the three pancreatic cancer cell lines and significantly reduce the cell number in dose and time dependent manner with IC_50_ of 100 μM (Fig. 1A). There was no cytotoxicity observed in HEK293 cells upon esculetin treatment (Additional file 1: Figure S1). Further, no reduction in viability was observed following the treatment of cells with vehicle (DMSO). Henceforth, all the assays were carried out on pancreatic cancer cells with 100 μM esculetin treatment, with appropriate controls.

To examine the antiproliferative potential of esculetin, an analysis of distribution of cells into different phases of cell cycle was done on esculetin treated and untreated cell culture. DNA histogram analysis, obtained after PI staining of esculetin treated and untreated pancreatic cell population, showed a significant increase in the percentage of cells in G1 phase in all the three cell lines (Fig. 1B, C). The increase in G1 phase cells was accompanied by a reduction in percentage of cells in S and G2/M phase. Thus treatment with 100 μM esculetin significantly reduced the growth of population of pancreatic cancer cells by arresting the cells in G1 phase.

### Esculetin induces apoptosis in pancreatic cancer cells by activation of caspases

**Fig. 2:**
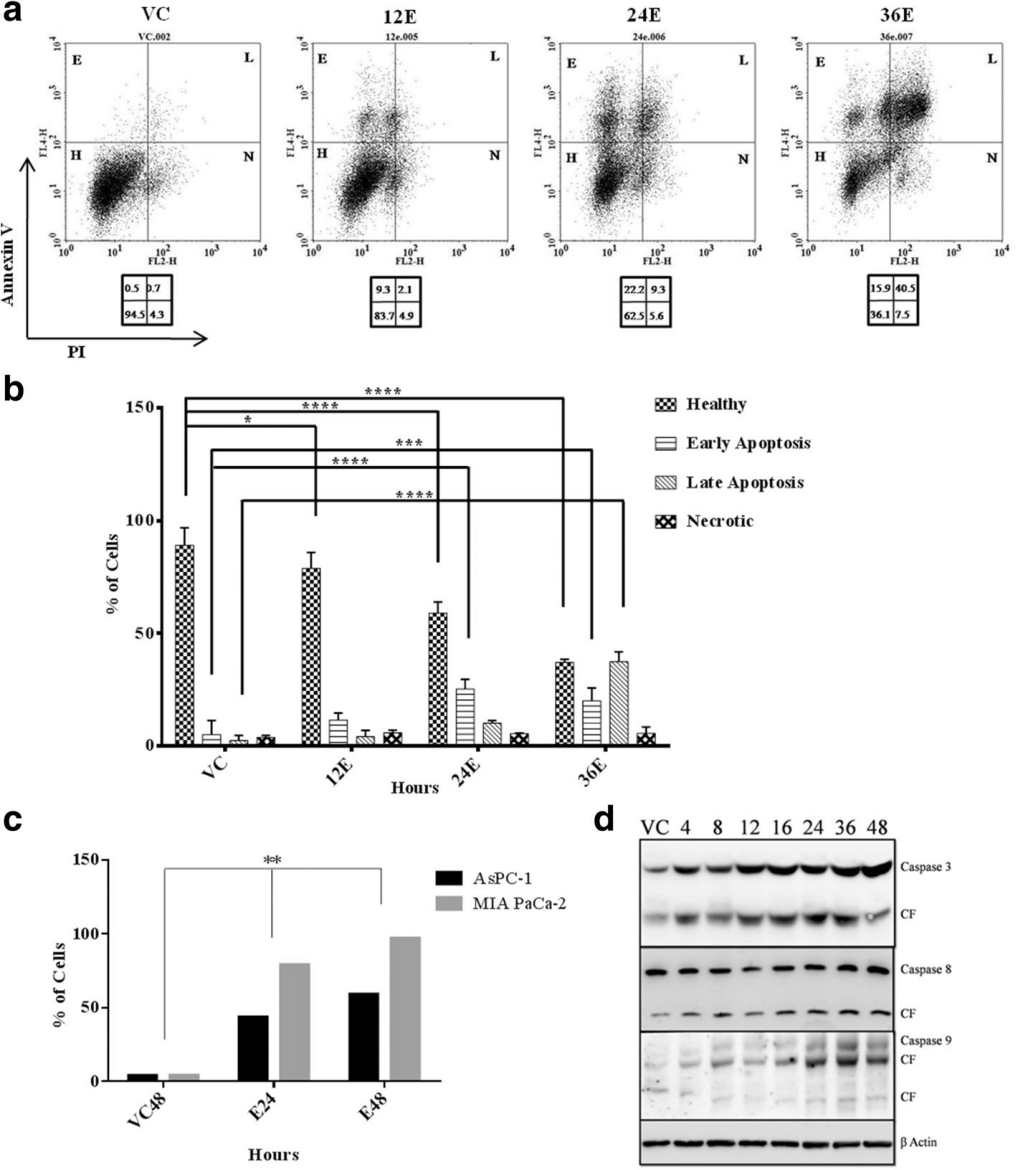
Esculetin induces apoptosis in pancreatic cancer cells: **a** Flow cytometric analysis of PANC-1 cells treated with 100 μM esculetin for indicated time showed temporal increase in surface expression of apoptotic marker- Annexin V indicating increased population of cells in apoptotic phase. **b** Percentage of PANC-1 cells exhibiting fluorescence in all four panels (healthy, early apoptosis, late apoptosis and necrosis) showing time dependent increase in apoptosis in Ecsuletin treated cells. **c** Percentage of cells with active APO BrdU indicating apoptosis in the absence and presence of esculetin (100 μM) as determined using TUNEL assay. **d** Western blot analysis showing an increase in expression of pro and active form of caspases (VC stands for vehicle control, E stands for esculetin treated sample for indicated time, CF stands for cleaved form, numerals represent time of esculetin treatment). Data represents the mean ± SD of three independent experiments. The significance was determined using ANOVA (Bonferroni’s test). Key:*p < 0.05; **p < 0.01; ***p < 0.001; ****p < 0.0001)

To check whether esculetin treatment is inducing apoptosis in cells, annexin-V-APC and PI cytometry assay was carried out in PANC-1 cells and APO-BrdU TUNEL Assay was carried out in MIA PaCa-2 and AsPC-1. A temporal increase in population of cells in early and late apoptosis could be observed in PANC-1 cells (Fig. 2a, b). By 36 h of esculetin treatment, more than 50 % of cells became apoptotic (Fig. 2a, b) and similar results were obtained in other two cell lines as well (Fig. 2c). It was then evaluated if apoptotic death was mediated by activation and cleavage of caspases. Western blot ananlysis revealed a temporal increase in expression of caspase 3,8 and 9 and their cleaved forms (Fig. 2d).

### Loss of mitochondrial membrane potential initiates apoptosis on esculetin treatment

**Fig. 3:**
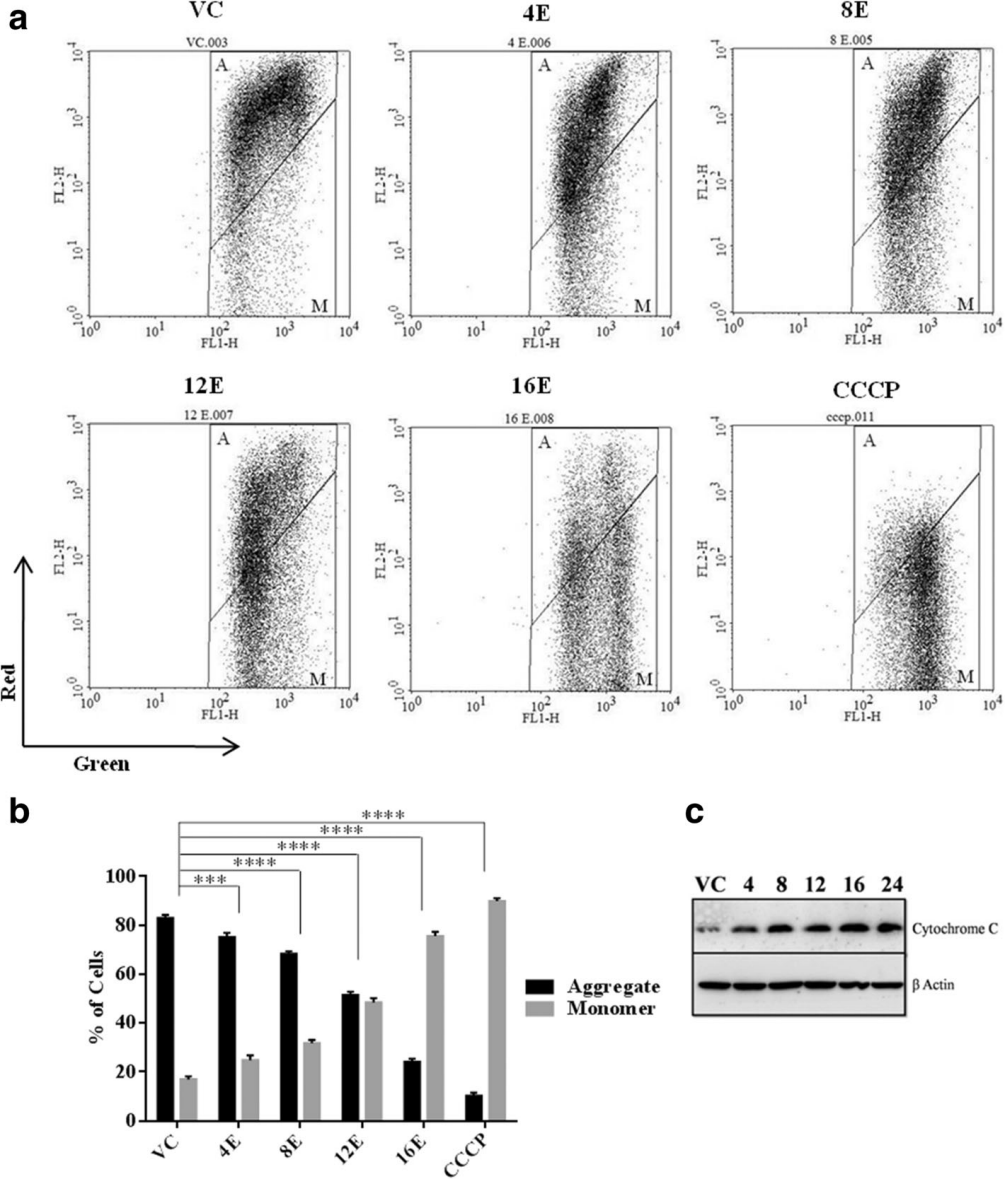
Esculetin induces loss of Mitochondrial membrane potential: **a** Flow cytometric analysis of PANC-1 cells stained with JC-1 dye after 100 μM esculetin treatment for indicated time showed a temporal decrease in ratio of *red* fluorescence (JC-1 aggregates) to *green* florescence (JC-1 monomers). **b** Percentage of cells exhibiting monomers and aggregates of JC-1 after treatment of cells with esculetin for different time intervals. **c** Western blot analysis showing temporal increase in cytosolic cytochrome C in PANC-1 cells. (VC stands for vehicle control, E stands for esculetin treatment sample for indicated time, CCCP stands for positive control i.e., carbonyl cyanide 3-chlorophenylhydrazone treated cells, numerals represent time of esculetin treatment). Data represents the mean ± SD of three independent experiments. The significance was determined using ANOVA (Bonferroni’s test). Key:*p < 0.05; **p < 0.01; ***p < 0.001; ****p < 0.0001)

The role of mitochondria in esculetin induced apoptosis in pancreatic cancer cells was investigated by examining change in status of mitochondrial membrane potential during treatment and determining the levels of cytosolic cytochrome C. In all the three cell lines, flow cytometric analysis of esculetin treated and untreated cells, stained with JC-1, presented a significant decrease in ratio of red (JC-1 aggregates) to green fluorescence (JC-1 monomers) on esculetin treatment (Fig. 3a, b; Additional file 2: Figure S2A,B,C). Further, western blot analysis of cytosolic protein displayed an increased level of cytochrome C in esculetin treated cells (Fig. 3c). The results thus confirmed loss of mitochondrial membrane potential in pancreatic cancer cells within 12 h of exposure to esculetin.

### Esculetin decreases intracellular ROS level and attenuates NF-κB

**Fig. 4:**
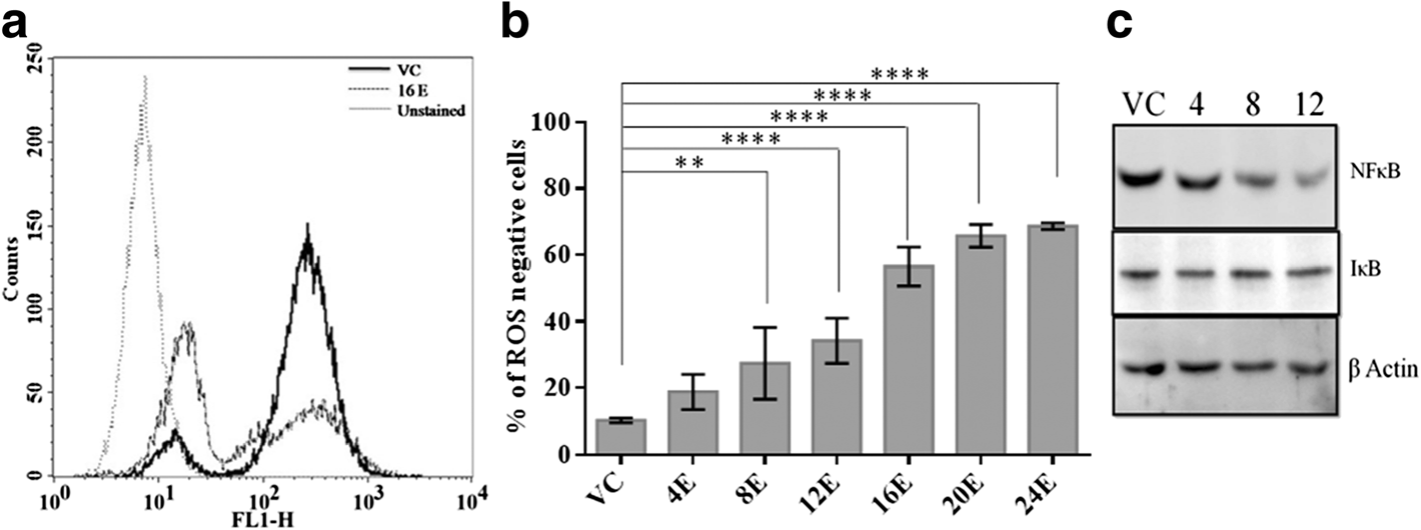
Esculetin lowers ROS levels: **a** Fluorescence intensity of DCFDA, an indicator of ROS level was determined in PANC-1 cells and it was found to decrease upon 100 μM esculetin treatment. **b** Flow cytometric analysis based histogram showing increase in percentage of PANC-1 cells negative for ROS activity as a function of time of ecsuletin treatment. **c** Western blot analysis showing temporal decrease in protein levels of ROS dependent transcription factor NK-κB and uniform levels of its inhibitor I-κB in PANC-1 cells. (VC stands for vehicle control, E stands for esculetin treated sample for indicated time, and numerals represent time of esculetin treatment). Data represents the mean ± SD of three independent experiments. The significance was determined using ANOVA (Bonferroni’s test). Key:*p < 0.05; **p < 0.01; ***p < 0.001; **** *p* < 0.0001)

To further explore the mechanism of action of esculetin, intracellular levels of ROS were determined in esculetin treated and untreated PANC-1 cells using DCFDA staining followed by flow cytometry. On exposure to esculetin, a conspicuous decrease in DCFDA fluorescence intensity, indicative of ROS levels, was observed in cells (Fig. 4a, b). The reduction could be seen as early as 4 h post esculetin treatment and by 16 h of treatment most of the population shifts to lower ROS level status. Further, the levels of p65 subunit of NF**-**κB, a ROS sensitive transcription factor, and its inhibitor I**-**κB were also determined by western blot analysis. The NF**-**κB protein levels were found to get decreased significantly within 8–12 h of esculetin treatment while those of I**-**κB remained unaltered (Fig. 4c).

### Esculetin activates ARE pathway in PANC-1 cells

**Fig. 5:**
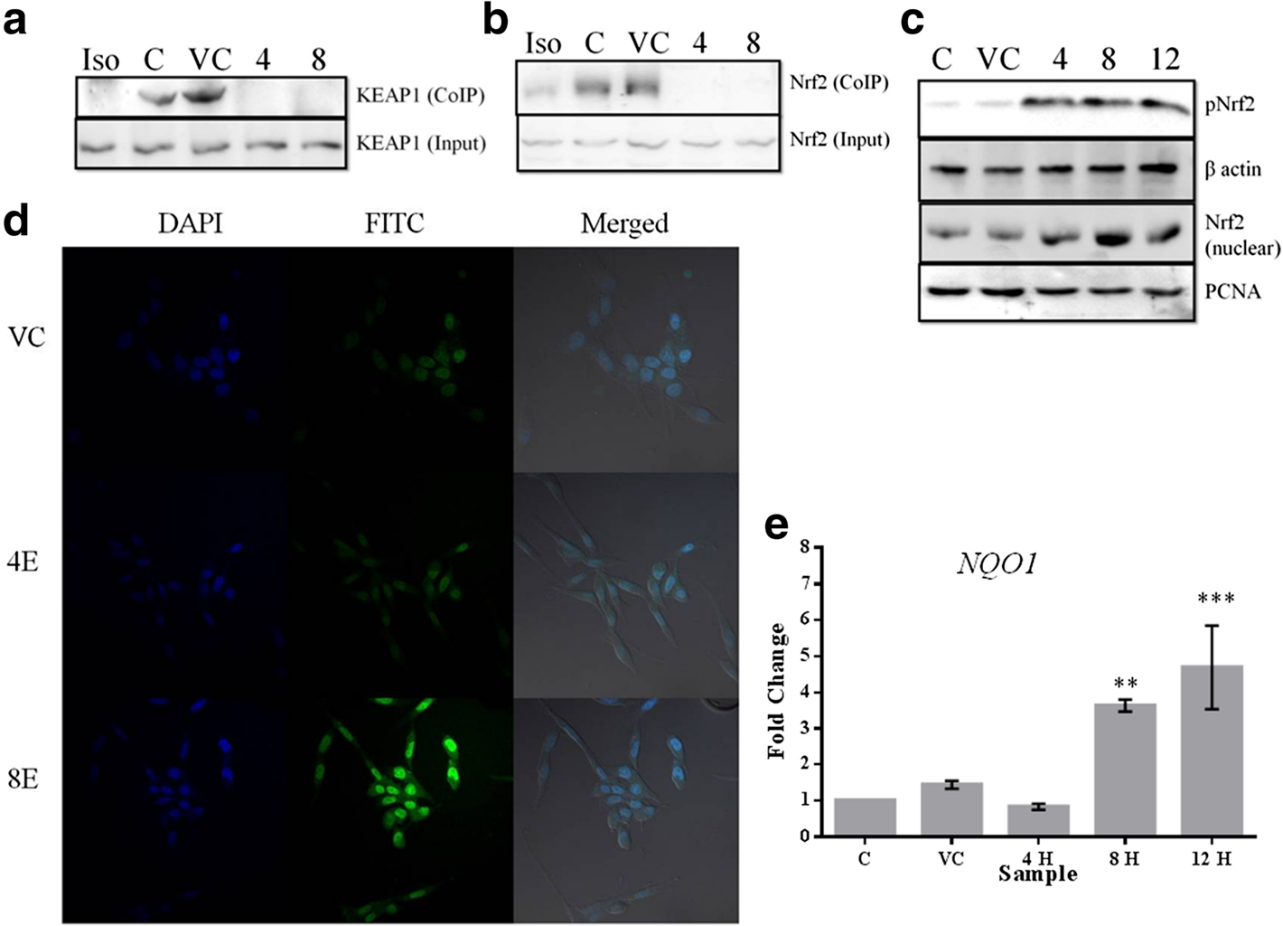
Esculetin disrupts Nrf2-KEAP1 interaction: **a** and **b** Western Blot analysis of PANC-1 protein extract immuno-precipitated using (**a**) Nrf2 and (**b**) KEAP1 antibody and probed with the other, showing loss of their interaction in esculetin treated cells. Input lane represents western blot analysis of 10 % of total protein extract used in CoIP indicating endogenous level of probed protein. **c** Western Blot analysis of total protein and nuclear extract from esculetin treated PANC-1 cells showing increase in phosphorylated form of Nrf2 and its nuclear accumulation. **d** Confocal microscopy view of esculetin treated PANC-1 cells incubated with Nrf2 antibody and probed with FITC (*green*) labeled secondary antibody showing an increase in nuclear accumulation of Nrf2. Nuclear staining was done with DAPI (*blue*). **e** Change in expression of NQO1, a target of Nrf2, measured using qPCR, showed about 5 fold increase upon 100 μM esculetin treatment. (C stands for control, VC stands for vehicle control, Iso stands for isotype Ab control, E stands for esculetin treated sample for indicated time). Data represents the mean ± SD of three independent experiments. The significance was determined using t test. Key:*p < 0.05; **p < 0.01; ***p < 0.001)

Once it became evident that esculetin lowers the level of ROS in PANC-1 cells, experiments were designed to unveil the pathway through which this response is mediated. To examine if esculetin affects the regulation of ARE pathway, the interaction between the key transcription factor of ARE pathway, Nrf2, and its inhibitor KEAP1 was inspected in the presence and absence of esculetin using Co-IP assay in PANC-1 and MiaPaCa2 cells. A remarkable loss of interaction could be seen by western blot analysis of immunoprecipitated protein in both the cell lines (Fig. 5a, b. Additional file 3: Figure S3). However, there was no change in total endogenous expression of both the proteins, as evident by western blotting of input protein. In contrast, an increase in the amount of phosphorylated form of Nrf2 was observed in esculetin treated cells, ascertaining the release of Nrf-2 from its inhibitor KEAP1 (Fig. 5c). Further, we observed an increase in the levels of Nrf2 in nuclear extract of esculetin treated cells indicating its nuclear accumulation (Fig. 5c). The localization of Nrf2 was also probed in vivo by confocal microscopy that asserted its nuclear accumulation on esculetin treatment (Fig. 5d). An increase (5 fold) in the expression of NQO1, a direct target of Nrf2, was also observed by qPCR in esculetin treated cells compared to that of untreated cells (Fig. 5e). Together these results establish that esculetin treatment activates ARE pathway pancreatic cancer cells due to disruption of Nrf2-KEAP1 interaction.

### Esculetin binds directly to KEAP1

**Fig. 6:**
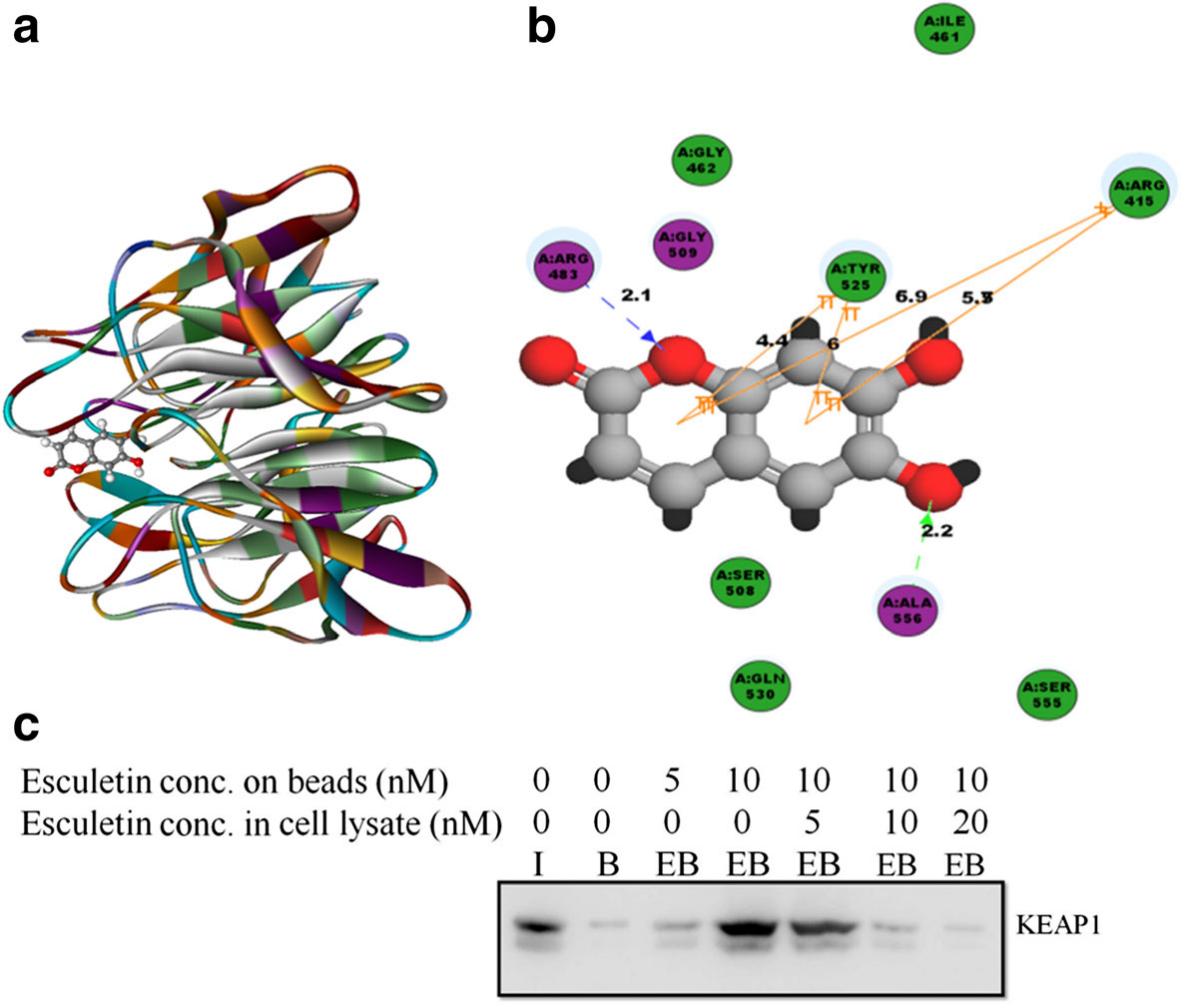
Esculetin binds to KEAP1 directly: **a** 3D Docking model showing esculetin docked to the active site of KEAP1. **b** 2D interaction diagram of esculetin with KEAP1. Residues involved in hydrogen-bonding, charge or polar interactions are represented by *magenta*-colored circles. Residues involved in van der Waals interactions are represented by *green* circles. The solvent accessible surface of a residue is represented by a *blue* halo around the atom. Hydrogen-bond interactions with amino acid side chain and main chain are represented by a *blue* and *green* dashed line, respectively with an arrow head directed toward the electron donor. π-π and π-cationic interactions are represented by an *orange* line with symbols indicating the interaction. **c** Western blot analysis of competitive pull down assay carried out with esculetin conjugated beads showing a direct interaction between esculetin and KEAP1. (I stands for input (10 %), B represents protein pulled using non conjugated beads, EB represent protein pulled using esculetin conjugated sepharose beads)

To explore if there exists a possible interacting mode of esculetin with KEAP1 (PDB code: 4IQK), molecular docking studies were performed using the docking program, CDOCKER with Discovery Studio 4.0. Esculetin was docked into the active site of the receptor using the ‘ligand fit’ function (Fig. 6a). The binding affinity between the esculetin and KEAP1 protein was calculated using the scoring function module of the Discovery Studio 4.0. The CDocker energy value for esculetin was observed to be −19.95 kcal/mol (Table 1). It was found to interact specifically with the P1 and P3 subpocket of KEAP1 receptor (Fig. 6b). The two aromatic rings of esculetin were observed to interact via a π-π interaction with Tyr525 with a bond distance of 4.4 Å and 6 Å respectively. Further, these rings were also observed to interact via a π-cationic interaction with Arg415 with a bond distance of 5.3 Å and 6.6 Å respectively. The compound was also found to be involved in the formation of hydrogen bond with the side chain of Arg483 and the main chain of Ala556, with a close distance of 2.1 Å and 2.2 Å, respectively. In addition, the molecule was stacked in a conformation that allows it to interact with Ser508, Ser555, Gln530, Gly462 and Ile461 residues via the hydrophobic interaction. Thus, these simultaneous binding of esculetin with the P1 subpocket of KEAP1 and the strong binding energy suggested the protein to be its direct binding target.

**Table 1:** Molecular docking scores of esculetin againt KEAP1

CDOCKER Energy	CDocker interaction energy	Lig Score 1	Lig Score 2	-PLP 1	-PLP 2	Jain	PMF	Ludi Score 1
−19.9531	−20.5527	2.37	3.63	39.12	35.66	−0.79	59.87	177

In the next step, Arg 415 and Arg 483 were replaced by alanine one by one in KEAP1 receptor and ligand esculetin was docked upon it. Significant changes were observed in the interaction and binding energy. The binding energy between esculetin and KEAP1 decreased to −15.9412 Kcal/mol in case of R415A mutation and to −16.6062 Kcal/mol in case of R483A mutation in the binding pocket. Further, most of the interactions previously observed with wild type KEAP1 were found to be lost in both mutants (Additional file 4: Figure S4A,B). It is therefore predicted that these two arginine residues are pivotal in the binding of esculetin to KEAP1.

To affirm the depicted binding between esculetin and KEAP1, a pull down assay was performed using esculetin conjugated Sepharose 4B beads with total cellular protein extracted from PANC-1 cells. The western blot analysis of the same clearly shows that the sepharose beads conjugated with esculetin (5nM and 10nM) could pull down much more KEAP1 protein than the unconjugated beads in a concentration dependent manner (Fig. 6c). To further confirm the specificity of binding, competition assay was performed using cell extract pre incubated with 5-20nM of esculetin before pull down. The mixture was then allowed to bind to affinity column (sepharose conjugated with esculetin) and the levels of KEAP1 protein now retained on the affinity column were checked by western blot analysis. Our results clearly indicated that pre-incubation of protein with increasing concentrations of esculetin resulted in a parallel decrease in retention of KEAP1 to the affinity column (Fig. 6c). The pull down assay thus unequivocally proved that esculetin binds directly to KEAP1.

### Pharmacokinetic profile of esculetin

To predict the drug-likeness of esculetin, a few indicators of their pharmacokinetic profiles were predicted using ADMET module, v. 4.0 (Accelrys Inc., San Diego, CA). Herein, the aqueous solubility, bloodbrain barrier penetration, cytochrome P4502D6 binding, hepatotoxicity, intestinal absorption, and plasma protein binding were calculated (Table 2). The ADMET hepatotoxicity probability for esculetin lies within range and thus doesn’t seem to possess any kind of hepatotoxic effects. Esculetin was predicted to be non-inhibitor of CYP2D6 enzyme and may be metabolized and excreted successfully. It was predicted to bind weakly to plasma proteins, so it will be available for diffusion or transport across cell membranes. Further, it showed high aqueous solubility and absorption levels which signifies high bioavailability of esculetin. The result thus suggests that esculetin has good physicochemical and pharmacokinetic profile.

**Table 2:** Predicted ADMET properties of the esculetin

Lipinski rule of five violations	PSA	BBB level	Absorption level	PPB level	Solubility level	CYP2D6	Hepatoxicity level
0	67.861	3	0	2	4	0	0

*BBB* stands for blood brain barrier, *PSA* stands for Polar surface area, *PBP* stands for plasma protein binding, *CYP2D6* stands for Cytochrome P450 2D6

PSA (60–70 Å2)

BBB level (0 very high, 1 high, 2 medium, and 3 very low)

Absorption level (0 good, 1 moderate, 2 low, and 3 very low)

PPB (0 very high, 1 high, 2 medium, and 3 very low)

Solubility level (4 very high, 3 high, 2 medium, and 1 very low)

CYP2D6 (0 non-inhibitor and 1 inhibitor)

Hepatoxicity level (0 is non toxic, 1 is toxic)

## Discussion

It is estimated that in the coming decade pancreatic carcinoma would be amongst the leading cause of death amongst cancer patients [26]. What makes this fact all the more alarming is that since 1997 there has been no improvement in the therapeutic regime for the same, which continues to be gemcitabine. The low median overall survival (6.7 months) of the patients, thus make the identification and development of new therapeutic compounds cardinal [27]. In our earlier studies we have demonstrated a plant driven coumarin compound, esculetin, to be a potential drug candidate for Acute Myeloid Leukemia with translocation (8;21) and mutation in C-Kit gene [13, 14]. In this study, to understand the mechanism of esculetin action, the antiproliferative and apoptotic effect was first established in pancreatic cell lines (PANC-1, MIA PaCa-2 and AsPC-1). We observed that esculetin treatment could impede the growth of PANC-1, MIA PaCa-2 and AsPC-1 cells in a dose as well as time dependent manner with IC 50 determined to be 100 μM. This IC50 value of esculetin in pancreatic cells is appreciably lower than its IC50 values determined in certain other cells- human leukemic cell line U937 (approximately 200 μM) [7]; human colon cancer cells HCT116 (approximately 600 μM) [28] and human hepatocellular carcinoma cells SMMC-7721 (approximately 2000 μM) [12]. Further, we found esculetin exposure arrested the cells in G1 phase, which seems to be its probable mechanism of action. Earlier reports too reported an increase in population of cells in G1 phase in various cell lines [14, 28, 29]. However in hepatocellular carcinoma, esculetin was reported to cause an S phase arrest. Thus it is definite that the compound would target pathways differentially depending upon the cells making it obligatory to investigate the mechanism followed in different cell types.

Most of the anticancer drugs eventually kill a cancerous cell by induction of apoptosis. Esculetin too has been reported to activate mitochondrial apoptotic pathway in cancer cells. In our study we could detect an increase in active caspases in esculetin treated cells. There was a marked increase in apoptotic markers on the surface of esculetin treated cells. In consistence with previous studies done on apoptosis induction by esculetin [12], we too observed loss of mitochondrial membrane potential in esculetin treated pancreatic cancer cell lines. The increase in cytosolic levels of cytochrome C explains well the activation and cleavage of caspase 3 and 9 observed by western blot analysis. However a similar elevation in cleavage of caspase 8 indicates activation of extrinsic pathway of apoptosis as well. Thus our studies on pancreatic cancer cells show that esculetin induces apoptosis in these cells through both extrinsic and intrinsic pathways.

A characteristic feature of cancer cells is an elevated ROS level which is attributed to excessive metabolic activity and damaged mitochondrial function. Further, ROS is required for cell cycle progression, inflammation, tumor growth and metastasis [21]. This rationalizes the recent development in ROS abrogating strategies to kill cancer cells. In our study, esculetin, an antioxidant, was speculated to mediate its effect in cancer cells through ROS modulation. As anticipated, we could perceive a decrease in ROS levels in PANC-1 cells on esculetin treatment. Various antioxidant agents have been shown to suppress tumor cell growth and in pancreatic cancer cells particularly, ROS production has already been shown to promote cell survival [30]. In understanding thoroughly the potential pathway pursued by esculetin, we checked the change in the levels of ROS sensitive transcription factor NF-κB in esculetin treated cells and we could detect an explicit decline in the same. Enhanced activity of NF-κB has already been reported to be associated with increased cell survival and proliferation due to elevated expression of antiapoptotic protein Bcl-2 and Bcl-xl as well as c-Myc and cyclin D1 that promotes progression of cell cycle from G1 to S phase [31, 32]. Further, certain other antioxidant agents like curcumin and other polyphenols and N-acetyl-L-cysteine (NAC) have been also found to impede cell cycle progression and promote apoptosis by inhibiting NF-κB activity [32–36]. Based on our results, and the supporting literature, we could remark that esculetin too produce anticancer response in PANC-1 cells by scavenging ROS and attenuating NF-κB activity. We also observed that the levels of its inhibitor (I-κB) remain the same upon esculetin treatment.

In an endeavor to identify the direct target of esculetin important leads from the results hitherto suggested scrutinizing the pathway responsible for declined ROS levels in esculetin treated PANC-1 cells. Further, inhibition of NF-κB by several antioxidants correlates with their redox capabilities and strong interplay between NF-κB and Nrf2 have been reported [36–40]. We thus explored the effect of esculetin treatment on the regulation of Nrf2, the master regulator of antioxidant activity. Nrf2, which combats the oxidative stress in a cell as an important anti-neoplastic factor and is emerging as a key target of cancer chemoprevention and chemotherapy [41]. Much the same as role of ROS, the role of Nrf2 is also debatable in cancer. Cancer cells have a tendency to hijack the regulatory machinery of Nrf2, whereby they maintain their intracellular ROS level within the range required for their proper growth and survival [42]. Basal expression levels of Nrf2 have been reported to be low in PANC-1 while that of it’s inhibitor KEAP1are reported to be high [43]. Although strategies inhibiting Nrf2 have gained focus as anticancer therapy [44, 45], yet its tumor suppressor role cannot be negated and there are still a number of studies focusing on developing small molecules that could inhibit Nrf2-KEAP1 interaction [17, 46–51]. Naurally occurring compounds like curcumin, lycopene, sulforaphane, oganosulfur compounds in garlic etc have been reported as potent activators of Nrf2 by inhibiting KEAP1, which has been linked to their chemopreventive and chemotherapeutic effects [51–53]. Further, synthetic compounds including triterpenoids derivatives like Bardoxolone methyl [RTA 408, methyl ester of cyano-3,12-dioxooleana-1,9(11)- dien-28-oic (CDDO- Me] too are being developed as anticancer agents along the same line [23, 54]. Such inhibitors would ultimately tend to decrease the intracellular ROS levels below the threshold required for cancer cells to proliferate. We too hereby provide evidence that there is a decreased interaction of Nrf2 with its inhibitor KEAP1 in esculetin treated cells whereas their endogenous levels remain unaltered. Upon release, Nrf2 is phosphorylated and transported to the nucleus wherein it binds to promoter with ARE sequence and activates the target genes encoding proteins that scavenge ROS and poses antagonistic effect on NF-κB pathway. Thus we propose that in pancreatic cancer cells there is a loss of interaction between Nrf2 and KEAP1 on exposure to esculetin that ultimately depletes ROS and abrogate NF-κB activity.

Shepherded by the already established leads, we excogitated to check if esculetin could bind directly to KEAP1. To ascertain the same, both *in silico* and in vitro approaches were followed. The binding cavity of KEAP 1 is divided into 5 subpockets as P1, P2, P3, P4 and P5, where two subpocket, P1 and P2, play significant role in strong binding interactions and contribute to total binding free energy. The P1, formed by residues Ser508, Phe478, Ile461, Arg483, Arg415, and Gly462, is highly positively charged and the electrostatic interactions with the Arg483 and Arg415 are significant for binding. The P2 subpocket, consisting of Ser363, Arg380, Asn382, and Asn414, is also positively charged whereas Arg415, located at the border of the P1 and P2, may take part in the P1 and P2 simultaneously. The peptide backbone occupies the P3 subpocket which is composed of Gly509 Ala556, Ser555, Ser602, Gly603, and Gly571 [22]. Molecular docking studies predicted significant hydrogen bond, π–cationic and π–π aromatic interactions between esculetin and residues in the subpocket P1 and P3 of the KEAP1 receptor, with a CDocker energy value of −19.95 kcal/mol. It was worth noticing that KEAP1 residues Arg 415 and Arg 483 were found to be involved in interaction with esculetin in our study, which have been reported in structural studies to be involved in Nrf2-KEAP1 binding by forming hydrogen bonds with Glu 78 of Nrf2 [55]. Mutagenesis experiments have provided strong evidences that individual substitution of these two amino acids in KEAP1 impaired its ability to bind with Nrf2 and to mediate repression of Nrf2-dependent gene expression [56, 57]. Herein, we also observed that alanine substitution of these residues in molecular docking experiments reduced the binding potential between esculetin and KEAP1 indicating their importance in KEAP1-esculetin interaction. Thus it is evident that both the interactions, Nrf2-KEAP1 and KEAP1-esculetin, involve same residues and apparently esculetin competes with Nrf2 for binding with KEAP1. Binding between esculetin and KEAP1 was further confirmed in vitro with pull down assay using esculetin conjugated beads. The variation in the amount of KEAP1 pulled down, an increase with increase in amount of esculetin coupled with beads and a decrease with increase in concentration of competing free esculetin, makes it evident enough that esculetin binds directly to KEAP1. The existence of this direct binding of esculetin to KEAP1, involving the same residues of KEAP1 as involved in its interaction with Nrf2, along with observed loss of Nrf2-KEAP1 interaction in pancreatic cancer cells upon esculetin treatment stipulates that esculetin perturbs the binding between Nrf2 and KEAP1.

An important requisite for a promising candidate to be developed as a drug is an investigation of its pharmacological properties. ADMET studies are expected to reduce the risk of late-stage attrition of drug development. Given the fact that esculetin has antiproliferative effects on cancer cells, it justifies itself to be screened for its drug likeness and our results strongly support esculetin to be a potential drug candidate.

## Conclusion

To conclude we can assert that esculetin is a good potential therapeutic agent for pancreatic cancer, where it activates the Nrf2-ARE pathway by binding to KEAP1 protein and ultimately suppresses cancer cell population growth through ROS sensitive transcription factor NFκB. Further studies should be carried out using animal models to check its efficacy and toxicity.

## Additional files

Additional file 1: Figure S1. Effect of esculetin on HEK293 cancer cells: Effect of different concentrations of esculetin on cell viability using MTT assay in HEK 293 cell line. Data represents the mean ± SD of three independent experiments. (TIF 23 kb)
Additional file 2: Figure S2. Esculetin induces loss of Mitochondrial membrane potential: A- Flow cytometric analysis of MiaPaCa-2 cells stained with JC-1 dye after 100 μM esculetin treatment for indicated time showed a temporal decrease in ratio of red fluorescence (JC-1 aggregates) to green florescence (JC-1 monomers). B,C- Percentage of MiaPaCa-2 cells (B) and AsPC-1 (C) exhibiting monomers and aggregatesof JC-1 after treatment of cells with esculetin for different time intervals. (VC stands for vehicle control, E stands for esculetin treatment sample for indicated time, CCCP stands for positive control i.e., carbonyl cyanide 3-chlorophenylhydrazone treated cells, numerals represent time of esculetin treatment). Data represents the mean ± SD of two independent experiments. The significance was determined using ANOVA (Bonferroni’s test). Key:**p* < 0.05; ***p* < 0.01; ****p* < 0.001; *****p* < 0.0001). (TIF 294 kb)
Additional file 3: Figure S3. Esculetin disrupts Nrf2-KEAP1 interaction: Western Blot analysis of MiaPaCa-2 protein extract immuno-precipitated using Nrf2 antibody and probed with KEAP-1, showing loss of their interaction in esculetin treated cells. Input lane represents western blot analysis of 10 % of total protein extract used in CoIP indicating endogenous level of probed protein. (C stands for control, VC stands for vehicle control, Iso stands for isotype Ab control, E stands for esculetin treated sample for indicated time). (TIF 208 kb)
Additional file 4: Figure S4. Interaction between esculetin and mutant KEAP1: 2D interaction diagram of esculetin with (A) KEAP1 R415A and (B) KEAP1 R483A. Residues involved in hydrogen-bonding, charge or polar interactions are represented by magenta-colored circles. Residues involved in van der Waals interactions are represented by green circles. The solvent accessible surface of a residue is represented by a blue halo around the atom. Hydrogen-bond interactions with amino acid side chain and main chain are represented by a blue and green dashed line, respectively with an arrow head directed toward the electron donor. (TIF 164 kb)

## Abbreviations

ARE: Antioxidant response element
IκB: Inhibitor kappa B
KEAP1: Kelch-like ECH-associated protein1
*NFE2L2*: Nuclear factor-Erythroid 2-like2
NF-κB: Nuclear factor kappa-light-chain-enhancer of activated B cells
NQO1: NAD(P)H:quinone oxidoreductase 1
Nrf2: Nuclear factor-Erythroid 2-related factor 2
ROS: Reactive oxygen species
