# Supplementary figures and images for: Esculetin induces antiproliferative and apoptotic response in pancreatic cancer cells by directly binding to KEAP1
Source: Mol Cancer. 2016 Oct 18;15:64. doi: 10.1186/s12943-016-0550-2 (PMC5069780; doi:10.1186/s12943-016-0550-2)

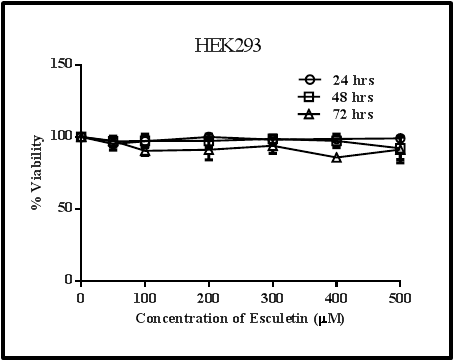

Supplement: Additional file 1: Figure S1. — Effect of esculetin on HEK293 cancer cells: Effect of different concentrations of esculetin on cell viability using MTT assay in HEK 293 cell line. Data represents the mean ± SD of three independent experiments. (TIF 23 kb) [file 12943_2016_550_MOESM1_ESM.tif]

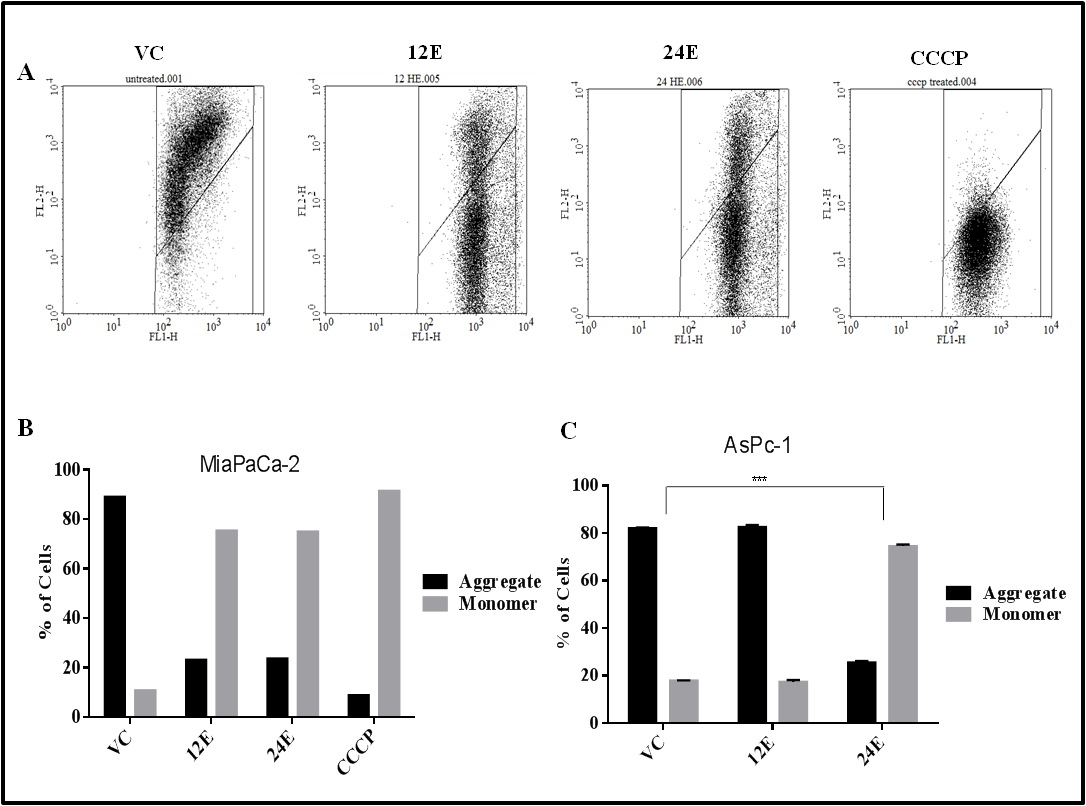

Supplement: Additional file 2: Figure S2. — Esculetin induces loss of Mitochondrial membrane potential: A- Flow cytometric analysis of MiaPaCa-2 cells stained with JC-1 dye after 100 μM esculetin treatment for indicated time showed a temporal decrease in ratio of red fluorescence (JC-1 aggregates) to green florescence (JC-1 monomers). B,C- Percentage of MiaPaCa-2 cells (B) and AsPC-1 (C) exhibiting monomers and aggregatesof JC-1 after treatment of cells with esculetin for different time intervals. (VC stands for vehicle control, E stands for esculetin treatment sample for indicated time, CCCP stands for positive control i.e., carbonyl cyanide 3-chlorophenylhydrazone treated cells, numerals represent time of esculetin treatment). Data represents the mean ± SD of two independent experiments. The significance was determined using ANOVA (Bonferroni’s test). Key:*p < 0.05; **p < 0.01; ***p < 0.001; ****p < 0.0001). (TIF 294 kb) [file 12943_2016_550_MOESM2_ESM.tif]

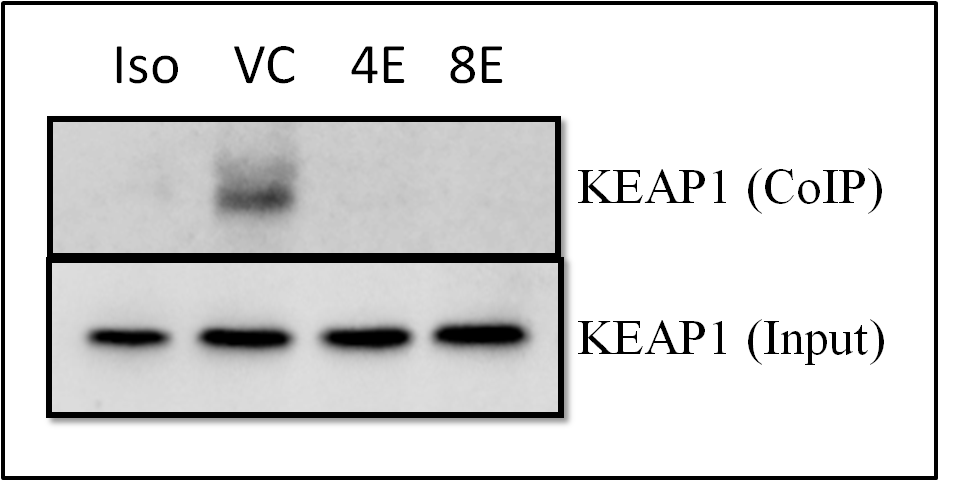

Supplement: Additional file 3: Figure S3. — Esculetin disrupts Nrf2-KEAP1 interaction: Western Blot analysis of MiaPaCa-2 protein extract immuno-precipitated using Nrf2 antibody and probed with KEAP-1, showing loss of their interaction in esculetin treated cells. Input lane represents western blot analysis of 10 % of total protein extract used in CoIP indicating endogenous level of probed protein. (C stands for control, VC stands for vehicle control, Iso stands for isotype Ab control, E stands for esculetin treated sample for indicated time). (TIF 208 kb) [file 12943_2016_550_MOESM3_ESM.tif]

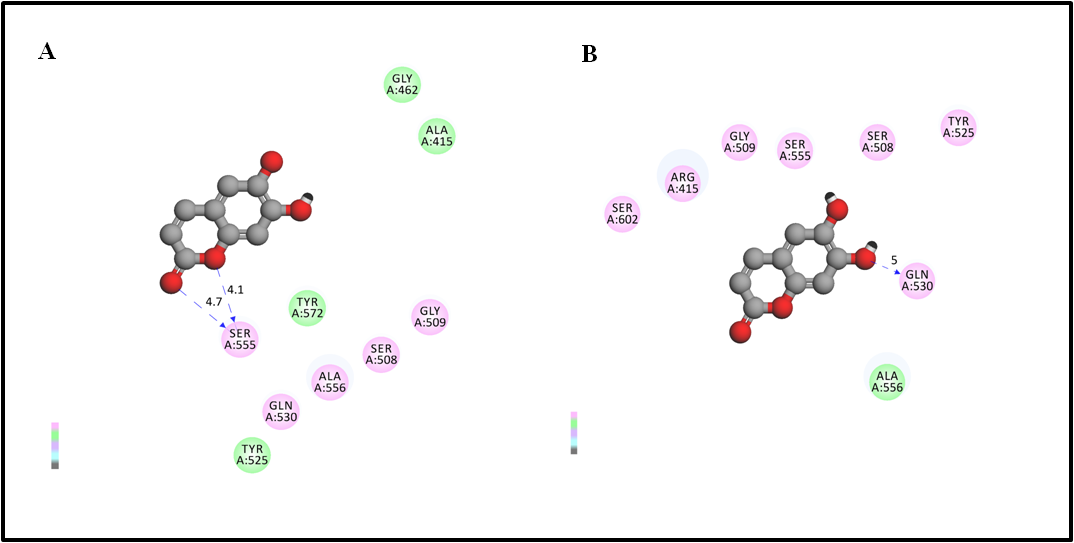

Supplement: Additional file 4: Figure S4. — Interaction between esculetin and mutant KEAP1: 2D interaction diagram of esculetin with (A) KEAP1 R415A and (B) KEAP1 R483A. Residues involved in hydrogen-bonding, charge or polar interactions are represented by magenta-colored circles. Residues involved in van der Waals interactions are represented by green circles. The solvent accessible surface of a residue is represented by a blue halo around the atom. Hydrogen-bond interactions with amino acid side chain and main chain are represented by a blue and green dashed line, respectively with an arrow head directed toward the electron donor. (TIF 164 kb) [file 12943_2016_550_MOESM4_ESM.tif]
